# Diabetes Diagnosis, Social Support, and Health Behavior Changes in Older Adults

**DOI:** 10.1093/geroni/igab046.410

**Published:** 2021-12-17

**Authors:** Weidi Qin

**Affiliations:** University of Michigan

## Abstract

The growing disease burden of diabetes in older adults highlights the importance of health-promoting behaviors in this population. A new diagnosis of diabetes can be a teachable moment that motivates older adults to engage in health behavior changes. Guided by the convoy model of social relations, social support from family and friends may influence health behaviors, and moderate the effects of a diabetes diagnosis on health behaviors. The current study investigates health behavior changes in drinking, smoking, and physical activity before and after a diabetes diagnosis, and whether social support moderates the relationships. A sample of 13,143 older adults without diabetes at baseline were selected from the Health and Retirement Study, and followed up for six waves. Social support from family and friends were measured separately. Mixed-effects regression models were performed. Sampling weights were adjusted to generate population estimates. After a diabetes diagnosis, older adults reduced alcohol consumption and were more likely to quit smoking. More social support from family was associated with decreased alcohol consumption, and more social support from friends was associated with increased physical activity. Significant interaction between social support from family and diabetes diagnosis was found. Specifically, among older adults with a diabetes diagnosis, more social support from family was associated with less drinking and smoking cessation. The study findings suggest that health practitioners can consider the timing of diabetes diagnosis to facilitate health behavior changes. Furthermore, diabetes educators can help older adults mobilize support from family and friends to better engage in health-promoting behaviors.

